# Clinical impact of real-time evaluation of the biological activity and degradation of hepatocyte growth factor

**DOI:** 10.1080/08977190802128083

**Published:** 2008-05-20

**Authors:** Fariba Nayeri, Tayeb Nayeri, Daniel Aili, Lars Brudin, Bo Liedberg

**Affiliations:** ^1^Division of Infectious Diseases, Faculty of Health Science, University Hospital, SE-58185 Linköping, Sweden; ^2^PEAS Institute, SE-58723 Linköping, Sweden; ^3^Department of Physics, Chemistry and Biology, Linköping University, SE-58183 Linköping, Sweden; ^4^Department of Clinical Physiology, Kalmar County Hospital, SE-38185 Linköping, Sweden; ^5^Department of Medicine and Health Sciences, University Hospital, Linköping, Sweden

**Keywords:** HGF, biological activity, SPR, chronic ulcer, inflammation

## Abstract

Hepatocyte growth factor (HGF) is essential for injury repair. Despite high HGF levels in chronic ulcers, up-regulation of HGF receptor in ulcer tissue and decreased biological activity of HGF in ulcer secretions have been observed. With a surface plasmon resonance-based method, we assessed the binding of HGF to antibodies, receptors, and the basement membrane and identified binding interactions that are indispensable for the biological activity of HGF. Recombinant HGF (rHGF) lots were tested for activity, structural integrity, and degradation, and the results were verified in an *in vitro* model of cell injury. Biologically active rHGF, as well as plasma from healthy volunteers, bound to heparan sulphate proteoglycan (HSPG) and to anti-HGF antibodies. Decreased binding to HSPG was the first event in rHGF degradation. This study established the feasibility of identifying patients with chronic inflammation who need exogenous HGF and of using ligand-binding assessment to evaluate rHGF lots for biological activity.

## Introduction

Hepatocyte growth factor (HGF) is a potent growth stimulator of mature epithelial cells that plays an important role in regeneration and repair after injuries (Matsumoto and Nakamura 1991). Natural HGF is a heterodimeric protein of 96–110 kDa that consists of one α chain (67 kDa) and one β chain (35 kDa; Godha et al. 1988). Mesenchymal cells produce HGF as a single-chain precursor (Arakaki et al. 1995), which is activated by the proteolytic function of HGF activator (Miyazawa et al. 1993). The HGF receptor, proto-oncogene c-Met, is a transmembrane protein expressed on epithelial cells (Prat et al. 1991). In addition, at least two binding sites for HGF on rat liver cell surfaces have been identified: a high-affinity c-Met/HGF receptor and a heparan sulphate proteoglycan (HSPG) binding site (Liu et al. 1992).

Basement membranes support epithelial and endothelial cells, prevent the passage of proteins, and generate histologically distinct compartments in the body. These membranes contain three major components: collagen IV, laminin, and HSPG, all of which are localized to the basal lamina portion of basement membranes. HSPG is a sulphated glycoprotein that binds and facilitates the cytokine–receptor interaction (Rescan et al. 1993) and the conversion of promitogen HGF to the two-chain form (Liu et al. 1992) High levels of HGF have been reported in blood during acute injuries such as infectious diseases (Nayeri et al. 2002c). HGF is also produced and released locally in a paracrine fashion at the site of acute injury (Nayeri et al. 2000, 2002b).

Surface plasmon resonance (SPR) is an optical technique that can simultaneously determine the affinity of a protein for several ligands by using individual surfaces on the sensor chip (Liedberg and Johansen 1998; Nayeri et al. 2005a). SPR enables rapid analyte detection in real time without any labelling of samples and can be used for concentration determination, kinetic studies, and epitope mapping. The sensor surface of the SPR apparatus consists of a carboxymethylated dextran matrix, which is a hydrogel that provides a solution-like environment for the biospecific interactions. The carboxyl groups in the dextran matrix enable the covalent coupling of ligands (proteins, receptors, DNA, etc.) to the surface. Upon testing several ligands that might bind HGF, we found at least four ligands that were important for distinguishing between different lots of rHGF or endogenous HGF (Nayeri et al. 2004, 2007). This difference might have biological consequences such as in the healing of ulcers (Nayeri et al. 2002d).

ELISA is a reliable, sensitive method for the detection of proteins in samples. Since 1998, we have determined the concentration of HGF indifferent recombinant batches or blood by using a commercial ELISA kit (R&D, Minneapolis, MN, USA), but we could not ascertain the epitope against which the monoclonal antibody in the kit was produced. However, we have obtained reproducible results with the HGF ELISA kit in several studies (Nayeri et al. 2000, 2002b,c). Whilst studying different lots of rHGF by ELISA, we observed that the concentration of rHGF in reconstituted samples correlated with the expected concentration. However, we did not find differences between biologically active and inactive lots by ELISA (Nayeri et al. 2004). Thus, the commercial ELISA kit determined the total amount of HGF but provided no information about the quality or activity. In a recent study, we showed that patients with chronic ulcers had HGF with decreased activity compared to patients with acute ulcers (Nayeri et al. 2004). This observation led us to investigate the quality of HGF both endogenously and in exogenous lots in *in vivo* (Nayeri et al.2007) and *in vitro* contexts.

In the present work, we introduce an SPR-based method to evaluate the quality and biological activity of rHGF according to the relative binding response of the cytokine to a number of biologically relevant ligands. The results obtained by this method correlated with high sensitivity and specificity to the results of a biological assay in the CCL-53.1 cell line, which responded specifically to HGF (Nayeri et al. 2007). The predominant binding response in biologically active HGF was identified. We also show that systemic HGF in the blood of patients with chronic leg ulcers had decreased quality compared with that of healthy controls.

## Materials and methods

### Patients

Venous blood was gathered from 16 patients with chronic leg ulcers (age range, 42–87 years; median age, 77 years; 4 men). Some of these patients were included in previous studies (Nayeri et al. 2004, 2005b). The plasma was separated and kept frozen at −70°C pending analysis.

### Ulcer secretions

Ulcer secretions were obtained from 10 patients with chronic leg ulcers and from 5 patients with acute ulcers. This material was used in previous studies from our group (Nayeri et al. 2004, 2005b).

### Healthy controls

Plasma was obtained from 20 people (age range, 34–83 years; median age, 55 years; 10 men) without any signs of infection and with no history of chronic leg ulcers and kept frozen.

### Recombinant human hepatocyte growth factor

Recombinant hHGF was a gift from Professor Nakamura, Osaka, Japan (2002). HGF was also obtained commercially (10 lots from R&D Systems, expressed in *Sf* 21 insect cells and in mouse myeloma cell line NSO; 3 lots from Sigma Aldrich, St Louis, MO, USA; 1 lot from Santa Cruz Inc., Santa Cruz, CA, USA; and 1 lot from GenWay Biotech Inc., San Diego, CA, USA).

### Buffy coat

A blood sample was obtained by venous puncture from a healthy blood donor, a 59-year-old man. The buffy coat was divided into two parts. The first portion was centrifuged at 3000*g* for 20 min, and the supernatant was collected. The other part was frozen at −70°C for 1h to haemolyse the blood cells, thawed, and centrifuged at 3000*g* for 20min, and the supernatant was collected. From the same individual, a blood sample was gathered in a tube (VENOJECT® silicone-coated) and centrifuged at 3000g for 20 min to collect the serum. Amounts (ELISA) and binding character (Biacore) of HGF in the buffy coat before and after haemolysis as well as in the serum were determined.

### Endogenous HGF

Venous blood from a healthy 55-year-old man was gathered in sterile silicone-coated tubes (VENOJECT®) and placed at room temperature for 1h. The liquid on top of the coagulated blood was gathered and added to Kaighn's modification of Ham's F-12K medium (ATCC) supplemented with 10% foetal bovine serum (Sigma-Aldrich, Stockholm, Sweden) in an atmosphere of 5% CO_2_ and 95% air at 37°C. After 48 h, the medium was centrifuged at 3000g for 20 min, and the supernatant was collected and kept frozen at −70°C.

### Evaluation of biological activity of HGF in a model of cell injury

The biological activity of HGF in samples was tested in an *in vitro* cell injury assay using transformed mouse skin epithelial cells (CCL-53.1 cell line). The method is described in a previous publication (Nayeri et al.2007). CCl-53.1 cells were grown in Kaighn's modification of Ham's F-12K medium (ATCC) supplemented with 15% horse serum and 2.5% foetal bovine serum (Sigma-Aldrich) in an atmosphere of 5% CO_2_ and 95% air at 37°C. After the cells reached confluence, they were separated with non-enzymatic cell dissociation solution (1 × )(Sigma-Aldrich), suspended in F-12K medium with 15% horse serum and 2.5% foetal bovine serum, and inoculated on a 24-well culture plate (Nunc Brand Products, Roskilde, Denmark). Cells were cultured under the same conditions for 24–48h until they reached confluence. Then, the confluent monolayer was scraped with a sterile steel device. Detached cells were washed with PBS, and fresh medium was added to the wells. The area of the square not covered by cells (mm^2^) was measured by microscopy (Olympus) and documented in each well. HGF was added, and the cells were incubated at 37°C in a humidified atmosphere containing 5% CO_2_. After 24 and 48h, the area not covered by a monolayer was measured again and documented.

### Ligands

Biologically relevant ligands of HGF [monoclonal anti-HGF antibody(unknown epitope), polyclonal antibodies against different parts of HGF, and HGF receptors] were obtained commercially (Table I) and immobilised on Biacore CM5 chips.

**Table I:** Ligands used to investigate the binding response of HGF by SPR.

Immobilised ligands in SPR	Source/product number	Code	Goal of investigation
Monoclonal anti-HGF AB	R&D Systems/RDS MAB294	MN	Determine amount of HGF
Recombinant HGF receptor (c-met)/fc chimera	R&D Systems/358 MT	c-Met	Analyze HGF binding to c-met receptor
HS proteoglycan	Sigma-Aldrich/H4777	HSPG	Analyze HGF binding to HSPG
Polyclonal anti-HGF AB, affinity isolated	Sigma-Aldrich/HH0652	PK	Determine amount of HGF
H-170 rabbit polyclonal AB	Santa Cruz/sc-13087	H-170	Bind amino acids 1–170 of human HGFβ
N-19 affinity-purified goat polyclonal AB	Santa Cruz/sc-1356	N-19	Peptide mapping at the N-terminus of human HGFβ
N-17 affinity-purified goat polyclonal AB	Santa Cruz/sc-1357	N-17	Peptide mapping at the N-terminus of human HGFα
C-20 affinity-purified goat polyclonal AB	Santa Cruz/sc-1358	C-20	Peptide mapping at the C-terminus of human HGFα
H-145 rabbit polyclonal AB	Santa Cruz/sc-7949	H-145	Bind amino acids 32–176 of human HGFα
D-19 goat polyclonal IgG	Santa Cruz/sc-34461	D19	
			Epitope mapping in an internal region of human HGFβ

All ligands were diluted (1:10 in 10 mM acetate buffer, pH 4.5) prior to immobilisation. AB, antibody.

### SPR measurements and ligand immobilisation procedures

SPR measurements were conducted at 760 nm in a fully automatic Biacore 2000 instrument (GE-Healthcare GmbH, Uppsala, Sweden) equipped with four flow cells. The flow cell temperature was 25°C in all experiments. The sample surfaces were carboxymethylated dextran CM5 chips (GE-Healthcare GmbH). Coupling of ligands to the carboxylic acid groups of the dextran hydrogel was carried out by conventional carbodiimide chemistry using 200 mM *N*-ethyl-*N*^′^-(3-diethylaminopropyl) carbodiimide (EDC) and 50 mM *N*-hydroxysuccinimide (NHS). The activation time was 7 min, followed by a 7-min ligand injection. Deactivation of the remaining active esters was performed by a 7-min injection of ethanolamine/hydrochloride at pH 8.5. A flow rate of 5 μl/min was used during immobilisation.

The ligands (Table I) were diluted in 10mM acetate buffer at a pH below the protein's isoelectric point, thus enhancing the electrostatic interactions between the dextran matrix and the ligands. The contact time was 7 min, which resulted in levels of immobilisation between 8000 and 30,000 response units (RU). After deactivation, the surfaces were washed with five subsequent 1-min injections of 5 mM glycine buffer, pH 2.0, with 1M NaCl (regeneration buffer). One of the flow cells was used to monitor the response due to interaction of HGF with the carboxymethylated dextran matrix. This flow cell was treated in the same way as the others during the immobilisation procedure, but the ligand immobilisation step was omitted.

Lyophilised rHGF was reconstituted and analysed at different concentrations. Sodium chloride (9 mg/ml, B. Braun Medical AB, Bromma, Sweden), distilled water(B. Braun Medical AB), phosphate buffer solution (pH 7.4, Apoteket AB, Umeå, Sweden), and HBS-EP (0.01 M HEPES, pH 7.4, 0.15 M NaCl, 3mM EDTA, 0.005% surfactant P20) (GE-Healthcare GmbH) were used as dilution buffers. A 1:1 mixture of 1M NaCl + 10 mM glycine, pH 2, was used as are generation buffer.

The blood samples were diluted 1:20 in phosphate buffer solution (pH 7.4, Apoteket AB). One injection of 1:1 1M NaCl:10 mM glycine, pH2, followed by one injection of borate 8.5 was used as a regeneration buffer for the blood samples. Leg ulcer secretions were diluted 1:10 in phosphate buffer solution. To avoid the effects of degrading enzymes, a protease inhibitor (1–5%) [4-(2-aminoethyl) benzenesulphonyl fluoride (AEBSF), pepstatin A, E-64, bestatin, leupeptin, and aprotinin (but no metal chelators); Sigma Aldrich] that specifically inhibited serine, cysteine, and aspartic proteases and aminopeptidases was added to the leg ulcer secretion 7–10 min prior to analysis. A1:1 mixture of 1M NaCl + 10 mM glycine, pH2, was used as a regeneration buffer.

The presented SPR data were extracted from the Biacore sensorgrams after the injections were finished, i.e. during the analyte dissociation phase. One Biacore RU corresponds to a surface concentration of 1 pg protein per mm^2^ (Stenberg et al. 1991). The binding of HGF to ligands was tested in fresh samples after reconstitution. The effect of time on the response was tested by continuous analysis of binding over time on the same chip and during the same run. The ligand binding of HGF after incubation at room temperature for 15 h was compared with ligand binding of a freeze-thawed sample. In addition, the effect of intracellular degrading enzymes on the binding of HGF to ligands was tested in buffy coat before and after haemolysis. A positive control was included at the beginning and at the end of each run to confirm the reliability of surfaces after several regeneration buffer injections.

### ELISA

A commercial ELISA kit (Quantikine Human HGF 96 tests, R&D Systems) was used to determine the concentration of HGF in samples. Our group has used this method since 1996, and the same person has performed all tests to reduce the inter- and intra-assay errors (Nayeri et al. 2000, 2002b,c).

### Statistics

Parametric (ANOVA) as well as non-parametric (Mann–Whitney *U* test, Spearman's regression test) (Statistica) tests were used for statistical analyses, and *p* > 0.05 was considered statistically significant.

## Results and discussion

The following procedures were repeated several times with different lots and had reproducible outcomes. Representative data are reported. The rHGF obtained as a gift from Professor Nakamura 2002 was biologically active and was SPR responsive (see below) and was used as positive control in some of the experiments. However in the reported results HGF produced by leukocytes in a healthy volunteer was used as a positive control.

### rHGF

#### Responsive lots

The rHGF lots that bound to all ligands (Tables I and II) with approximately the same absolute RU values (termed “SPR responsive”) had a motogenic effect on CCL-53.1 cells in the *in vitro* model of cell injury (>95% sensitivity and specificity). We showed previously (Nayeri et al. 2007) that the biologically active rHGF lots had motogenic effects on CCL-53.1 cells; therefore, the SPR responsive lots were considered biologically active (Table II). To simplify the calculation, we used the ratio of the binding responses to HSPG and to MN. Biological activity was observed in HGF lots in which the HSPG/MN ratio was ≥ 0.3. However, the cut-off ratio might be different in serum or plasma.

**Table II:** Analysis of SPR responsive rHGF

	MN (13065 RU)	HSPG (13092 RU)
5 μg/ml	991	741
2.5 μg/ml	644	421
1.5 μg/ml	250	110
0.6 μg/ml	150	49
0.3 μg/ml	63	14.3
0.1 μg/ml	25	4
HBS buffer	−4	0

Freshly reconstituted rHGF was diluted to different concentrations in HBS buffer, and the binding signal to two ligands was reported by SPR. This lot of rHGF was biologically active on CCL-53.1 cells. There was a significant positive correlation between the HGF concentration (μg/ml) and the binding signal to MN (SPR) in this lot (*r* = 0.98%, *r*^2^ = 0.97%, *p* < 0.0001). The immobilisation levels for ligands are given in parentheses.

In SPR responsive samples, there was a positive and significant correlation between HGF amount (μg/ml) and the SPR response to epitopes (*r*^2^ = 0.97, *p* < 0.0001). As shown in Table III, the binding signals for monoclonal anti-HGF (MN) and polyclonal anti-HGF (PK) decreased with dilution of the rHGF sample. A similar finding was obtained when we analysed HGF in faeces from patients with acute infectious gastroenteritis (Nayeri et al.2005a). In those patients, the SPR signal response correlated significantly with the amount of HGF determined by ELISA (*r* = 72%, *p* < 0.001). However, in another patient group with chronic inflammatory bowel disease, the SPR signal response did not correlate with ELISA results (article under preparation). These results might indicate that a positive correlation between ELISA and SPR is a determinant for biologically active HGF and that biologically active HGF is produced endogenously during acute inflammation.

**Table III:** SPR response (RU) and biological activity of samples containing HGF.

Samples containing HGF	c–met (22113 RU)	MN (19194 RU)	HSPG (14899 RU)	PK (20978 RU)	Nude area in mm after 24 h (reported as ratio of + HGF area/control well area)
5 μg/ml GJ175031	265	2000	226	326	nd
4 μg/ml GJ175031	201	1842	133	231	2.5/3.5
0.8 μg/ml GJ175031	120	473	77	132	0.5/3.2
0.8 μg/ml GJ175031 + PK	3.1	–1	–1	–3	3.5/3.5
0.08 μg/ml GJ175031	9	25	15	9	3.0/3.2
Endogenous HGF from blood donor	209	172	218	221	0.9/3.3
RHGF QF031062 2 – 100 ng/ml	12	6	−5	−3	3.4/3.5

The immobilisation levels for ligands are given in parentheses. The same chip was used for all measurements. Biologically active endogenous HGF was used as a positive control. The rHGF GJ175031 + PK as well as the biologically inactive rHGF lot GJ1750031 (Nayeri et al. 2005b) at different dilutions were used as negative controls. Because rHGF GJ175031 had binding affinity to all of the ligands in the study, this lot was termed “SPR responsive.” The biological activity was determined by measurement of the nude area after addition of rHGF to injured CCL-53.1 cells. The motogen activity of biologically active HGF causes migration of nearby cells towards the injured area and a subsequent decrease in nude area size (Nayeri et al. 2007). The biologically inactive rHGF QF031062 had no affinity to the ligands at different concentrations and was therefore “SPR non-responsive.”

**Figure 1:**
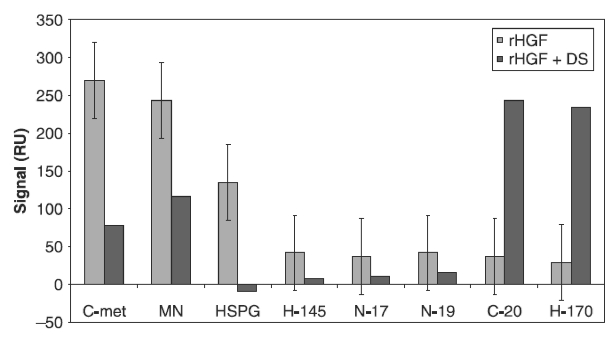
Effect of the addition of 10% DS (10 mg/ml in MQ water) to an rHGF lot (diluted in PBS) on the SPR binding response to different HGF ligands.

Binding of HGF to HSPG/dextran sulphate (DS) proved to be important for the biological activity of HGF. The addition of DS or HSPG to the samples reduced the binding (i.e. decreased the response) of HGF to the ligands (Figure 1), as well as decreased the biological activity of HGF. This observation is similar to the results obtained from analysis of HGF in the faeces of patients with acute infectious gastroenteritis (Nayeri et al. 2005a), in which the binding signal to HGF ligands in SPR decreased significantly after the addition of DS to the samples. The decrease in binding signal might imply that the dextran-binding site of HGF overlaps with the antibody-binding epitope, or that dextran binding induces a conformational change in the HGF molecule.

In common with many other growth factors (e.g. the fibro blast growth factor family and vascular endothelial growth factor), HGF displays a strong affinity for heparin, and this proved to be a useful aid in its purification (Nakamura et al. 1986). Heparan sulphate (HS) is widely expressed, in the form of various proteoglycan species, on the surfaces of most cells and is secreted directly into the extracellular matrix (Nakamura et al. 1986). Lyon et al. (1994) demonstrated by affinity chromatography that HGF interacted with HSPG under physiological conditions of pH and ionic strength. This interaction appeared to be mediated solely by the HS chains and was of comparable affinity to the interaction of HGF with heparin. In addition, heparin competitively inhibited the binding of HGF to HSPG. These results indicate that the primary HS-binding site, and perhaps also the heparin-binding site, is present on the α-chain of HGF (Gallagher 1989). The HGF-binding site is present within the iduronate- and sulphate-rich domains of HS, and its structure has been partially elucidated (Gallagher 1989). The interaction of HSPG with HGF seems to be mediated primarily by the HS chains and not the protein core.

Figure 1 shows that the binding of HGF to antibodies raised against the N-terminus of HGFβ (N19), the N-terminus of HGFα (N17), amino acids 32–176 of HGFα (H145), and an internal region of HGFβ (D19) might correlate with the dextran-binding affinity of HGF. The present work does not address the mechanism of the difference between biologically active and inactive HGF with regard to the binding affinity to these epitopes. However, previous studies have highlighted the role of HGFβ in the biological activity of HGF. Stamos et al. (2004) showed that the HGF β-chain alone binds c-Met and reported the crystal structure of HGFβ in complex with the Sema and PSI domains of the c-Met receptor. The c-Met Sema domain folds into a seven-bladed β-propeller in which the bottom faces of blades 2 and 3 bind to the HGF β chain “active site region”. Mutation of HGF residues in the area that corresponds with the active site region in related serine proteases significantly impaired HGFβ binding to c-Met. Lokker et al. (1992) reported that an HGF variant with mutations at residues Y673 and V692, which are located in the active site region of the HGF β-chain, had binding affinity for c-Met in the context of two-chain HGF but had significantly impaired biological activity. In a previous study, we showed that after the binding of HGF from conditioned medium of injured keratinocytes to the c-Met receptor, the affinity of HGF for the H-145 antibody (Table I) decreased, which might indicate that the H-145 epitope is involved in HGF interaction with the c-Met receptor (Nayeri et al. 2006). Further studies are planned to investigate the underlying mechanisms for these observations.

#### Non-responsive lots

The lots of rHGF that showed no binding to any of the ligands or that bound MN (Table I) but displayed much lower binding of HSPG had no biological activity in the CCL-53.1 cell line (Nayeri et al. 2007) and were therefore regarded as biologically inactive (Table II). Binding of HGF to HSPG is important to facilitate the contact between HGF and its receptor (Liu et al. 1992; Rescan et al. 1993). This factor might explain why the rHGF lots without affinity for HSPG and DS were biologically inactive.

**Figure 2:**
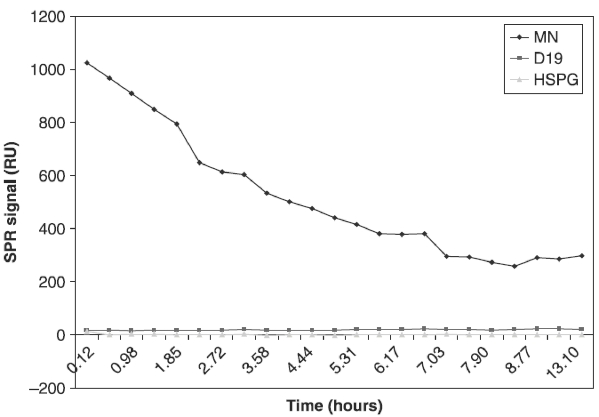
Degradation of protein with time in the same run. The diagram shows a simplified report of the Biacore run (SPR signal; RU) during 15 h in the same chip. rHGF carrier-free R&D (GJ1907011) was reconstituted in 2 ml PBS to a concentration of 2.5 μg/ml. The ratio of the binding response to HSPG/MN in this lot (rHGF carrier-free, R&D GJ1907011) was 14/1025 = 0.01; therefore, the lot was considered biologically inactive. Positive and negative controls were included in the study but are not shown.

Furthermore, the SPR non-responsive lots did not have the same affinities to antibodies developed against HGF peptides (Table I). In some lots there was a high affinity of HGF for MN (with unknown epitope, Table I) but a much lower affinity to other surfaces (Figure 2). In some lots there was no affinity to surfaces at all (Table III). As mentioned previously, the SPR responsive, biologically active lots showed a binding response to all surfaces. We have not studied the underlying mechanisms for such differences in ligand-binding profiles between SPR responsive and non-responsive lots. Further studies are planned to analyse the molecular basis of these differences.

#### Time-related changes in binding

By repeated analysis of the binding responses of different HGF lots to surfaces with time, we observed that the freshly reconstituted rHGF lots from the same batch had a similar binding response to ligands. However, this binding response decreased within minutes after reconstitution. Thus, we analysed the binding response of one SPR responsive and one SPR non-responsive lot to ligands that might be important in distinguishing the biologically active lots. As shown in Figure 2, the non-responsive rHGF lot had an initially high affinity to MN but much lower affinity to D19 and HSPG. However, the binding response to MN decreased significantly over time. On the other hand, in a reconstituted SPR responsive rHGF lot, the binding of HGF to HSPG decreased as the first step in the process of inactivation (Table IV). In another experiment, the binding of carrier-free HGF was better preserved after 15 h at room temperature than after thawing a sample within 15h of freezing (–20°C) and reconstitution (Table V). Although these are preliminary observations, the results might indicate that several freeze-thaw cycles are not beneficial for the biological activity of rHGF lots.

**Table IV:** Binding affinity of biologically active rHGF to MN and HSPG during incubation at room temperature.

GJ1750031	MN (22500 RU)	HSPG (14500 RU)
rHGF, 1-h incubation	309	105
rHGF, 24-h incubation	372	10
rHGF + DS, 1 h	123	25
rHGF + DS, 24 h	186	−17
rHGF + MN 1:20, 1 h	23	33
rHGF + MN, 24 h	−0.3	−24
rHGF original + HSPG 1:20	116	−30

Lyophilized rHGF (5 μg/ml) was reconstituted in PBS, pH 7.4, to 1ml (5000 ng/ml). 100 μl was added to one tube containing 900 μl PBS and to two other tubes containing 890 μl PBS and 10 μl DS or MN to reach the dilution of 1:10 (500 ng/ml). HSPG was added to 100 μl un-diluted rHGF (5000 ng/ml). The initial four samples were analyzed in one Biacore run. Dilution and running of the four samples took 1 h. The samples were kept at room temperature for 24 hand then re-analysed in the same chip. The immobilisation levels (RU) are given in parentheses.

**Table V:** Binding of recombinant HGF (R&D carrier-free GJ174034, diluted in PBS) to ligands, showing degradation over time.

Samples containing HGF	C-met (22377 RU)	MN (19820 RU)	HSPG (14337 RU)
2.5 μg/ml freshly reconstituted	194	1260	95
After 34 min + PK (10 μl)	1	−1	−19
2.5 μg/ml after 15 h room temperature	181	373	147
2.5 μg/ml after 15 h, −20°C and thawed	94	283	45
Negative control PBS	−10	−8	−24
Positive control after 15 h	403	508	512

The results are obtained from Biacore runs performed on the same chip. The immobilisation levels (RU) are mentioned in parentheses.

#### Assessment of quality

**Figure 3:**
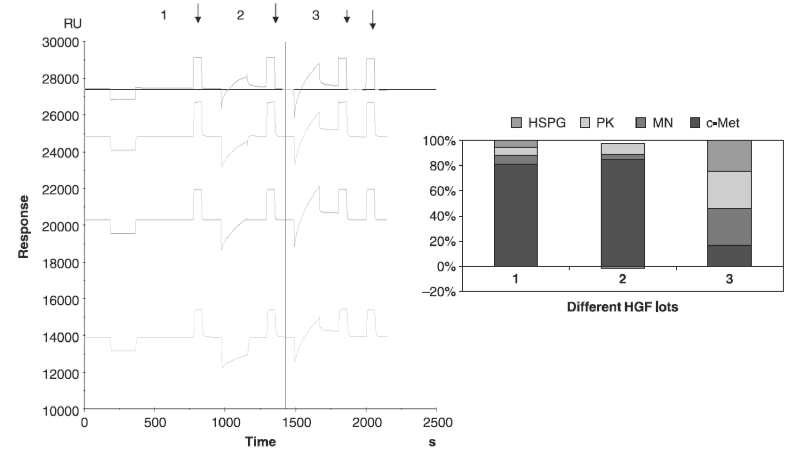
The histogram corresponding to a Biacore sensorgram from three lots of HGF. The first and second sensorgrams (1–2)(SPR non-responsive) belong to HGF with no biological activity in the *in vitro* assay (CCL-53.1 cells). The last sensorgram (3) belongs to biologically active HGF, which bound to all ligands with approximately the same absolute RU values (SPR responsive). The pills are showing the regeneration injections.

By using the SPR-based method, we observed differences between HGF lots in the relative ligand-binding responses that correlated significantly with their quality (Figure 3). Samples containing bacteria or chemical preparations, which are unsuitable for biological *in vitro* assays, could be evaluated for HGF quality by SPR. In some rHGF lots, the appropriate dilution of HGF to use in experiments could be determined by analyzing different concentrations of rHGF with the Biacore instrument and by choosing the concentration that gave the most similar binding to all ligands (Table III).

#### Buffy coat

rHGF lots were quite unstable in the SPR based analysis of HGF. However, our previous studies that analysed HGF in several patient groups (Nayeri et al. 2000, 2002b,c) and that investigated the stability of HGF in serum/plasma (Nayeri et al. 2002a) showed that endogenous HGF is fairly stable. The exact source of blood-borne HGF is unclear, but because HGF is present in many tissues, the tissues could contribute to plasma HGF in an endocrine manner. Another potential source of HGF in the blood is leukocytes, which contain substantial amounts of immuno reactive HGF (Wolf et al. 1991; Beppu et al. 2001).

We questioned whether chemical stress on the relatively stable endogenous HGF in blood might influence its binding affinity to the ligands that were important in distinguishing biologically active HGF. The buffy coat of peripheral blood is readily available from blood banks and contains a variety of white blood cells (Wang et al. 2006). Whole blood was gathered from a healthy volunteer, and the buffy coat was separated. The concentration (ELISA) and binding profile of HGF in the supernatant of buffy coat and in the serum of the same blood donor were analysed and shown to be similar (Table V). This result might indicate that serum is a reliable source for analysis of endogenous HGF. The pellet that contained the cells was placed at −70°C and then thawed to induce haemolysis. The concentration of HGF determined by ELISA in the haemolysed sample was higher than that in the non-haemolysed buffy coat, but the SPR response for all ligands in Biacore had decreased by, ≈50% (Table VI). This finding might indicate degradation of HGF by enzymes released after leukocyte lysis, which resulted in a high amount of biologically inactive HGF. Thus, SPR might be a more accurate indicator of protein quality than ELISA.

**Table VI:** Analysis of HGF in buffy coat of blood donor by SPR (RU) and ELISA (ng/ml).

	HSPG	c-Met	N-17	D19	ELISA (dilution 1:10)
Buffy coat centrifuged 3000g for 20 min, diluted 1:20 in PBS	35	247	272	335	3.7
Serum from same person, diluted 1:20 in PBS	38	317	336	330	2.3
Haemolysed buffy coat, frozen at -70°C, thawed, centrifuged at 3000g for 20 min, diluted in 1:20 PBS	15	163	99	118	5.7

Statistical analysis of results could not be performed because there were too few observations.

### Patients

The HGF binding profile in patients with chronic leg ulcers was examined. We had observed previously that these patients had high amounts of endogenous HGF in ulcer secretions but decreased biological activity of HGF (from the same sample) in the CCL-53.1 cell line (Nayeri et al. 2004, 2007). There was no significant correlation between age or sex of patients and healthy controls and the SPR response of HGF to the ligands. The binding response of HGF in plasma and in the ulcer secretions of patients with chronic leg ulcers differed significantly from that of healthy controls (Table VII). Binding to ligands crucial for the biological activity of HGF (e.g. HSPG, N17 and N19) was decreased in the plasma from patients with chronic ulcers compared with the plasma from healthy controls. However, direct biological activity of HGF was not observed upon adding whole blood, serum, or plasma to CCL-53.1 cells.

**Figure 4:**
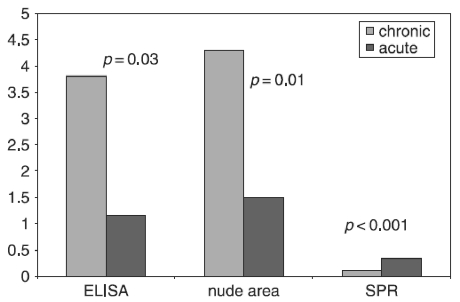
Concentration (detected by ELISA), biological activity (assessed in CCL-53.1 cells), and binding response to ligands (analysed by SPR) of HGF in ulcer secretions of patients with acute or chronic ulcers. The data on HGF concentration and biological activity were reported previously (Nayeri et al. 2004). The biological activity was determined by measurement of the nude area after addition of rHGF to injured CCL-53.1 cells. The motogen activity of biologically active HGF causes migration of nearby cells towards the injured area and a subsequent decrease in nude area size (Nayeri et al. 2007). In the present study, the ulcer secretions of the same patients and the same material were analysed by SPR. There was a significant negative correlation (*r* = 0.70, *r*^2^ = 0.49, *p* = 0.003) between the SPR binding response to ligands (HSPG/MN ratio) and the size of the nude area in injured CCL-53.1 cells.

The ratio of the binding response of HSPG/MN in leg ulcer secretions correlated significantly with the biological activity of HGF (Figure 4). Therefore, patients with chronic leg ulcers exhibited a change in the quality of HGF in the blood and decreased biological activity of HGF in ulcer secretions. However, the mechanism behind the changed quality of HGF in the blood of patients with chronic leg ulcers is unclear. In some other chronic inflammatory diseases such as lung fibrosis, increased levels but decreased biological activity of HGF have been reported (Yamanouchi et al. 1998; Marchand-Adam et al. 2006). Further studies are planned to investigate the mechanisms in patients with chronic leg ulcers.

**Table VII:** Statistical analysis of SPR ligand-binding response (RU) of endogenous HGF in plasma of patients with chronic leg ulcers (*n* = 16) compared with healthy controls (*n* = 20).

Ligands	Median chronic ulcers	Median control	*p* value
N-17	173	232	0.005*
HSPG	216	450	0.010*
HSPG	217	449	0.011*
N-19	220	446	0.014*
HSPG	222	444	0.017*
HSPG	224	442	0.021*
H-145	232	434	0.042*
H-170	245	421	0.10
C-20	251	415	0.15
PK	276	389	0.53
MN	279	386	0.60
c-Met	281	385	0.64

The ligands (Table I) were immobilised on four Biacore chips, and HSPG was included in all four chips. The *p* values (* = significant) indicate that the binding affinity of HGF to HSPG, N-17, N-19, and H-145 differed significantly between groups.

## Conclusion

SPR-based assessments of the binding profile of HGF to relevant ligands might rapidly and sensitively distinguish HGF variants with biological activity. Appropriate for clinical studies, this method might be used for evaluation of the quality of endogenous HGF and for recognition of patients who might benefitfrom exogenous HGF treatment.

## References

[b1] Arakaki N, Kawakami S, Nakamura O, Ohnishi T, Miyazaki H, Ishii T, Tsubouchi H, Daikuhara Y (1995). Evidence for the presence of an inactive precursor of human hepatocyte growth factor in plasma and sera of patients with liver diseases. Hepatology.

[b2] Beppu K, Uchiyama A, Morisaki T, Matsumoto K, Nakamura T, Tanaka M, Katano M (2001). Hepatocyte growth factor production by peripheral blood mononuclear cells of recurrent cancer patients. Anticancer Res.

[b3] Gallagher JT (1989). The extended family of proteoglycans: Social residents of the pericellular zone. Curr Opin Cell Biol.

[b4] Godha E, Tsubouchi H, Nakayama H, Hirono S, Sakiyama O, Takahashi K, Miyazaki H (1988). Purification and partial characterization of hepatocyte growth factor from plasma of a patient with fulminate hepatic failure. J Clin Invest.

[b5] Liedberg B, Johansen K, Rogers KR, Muchandani A (1998). Affinity biosensing based on surface plasmon resonance detection, methods in biotechnology. Affinity sensors: Techniques and protocols.

[b6] Liu K, Kato Y, Narukawa M, Kim DC, Hanano M, Higuchi O, Nakamura T, Sugiyama Y (1992). Importance of liver in plasma clearance of hepatocyte growth factor in rats. Am J Physiol.

[b7] Lokker NA, Mark MR, Luis EA, Bennett GL, Robbins KA, Baker JB, Godowski PJ (1992). Structure-function analysis of Hepatocyte growth factor: Identification of variants that lack mitogenic activity yet retain high affinity receptor binding. EMBO J.

[b8] Lyon M, Deakin JA, Mizuno K, Nakamura T, Gallagher JT (1994). Interaction of hepatocyte growth factor with heparan sulfate. Elucidation of the major heparan sulfate structural determinants. J Biol Chem.

[b9] Matsumoto K, Nakamura T (1991). Hepatocyte growth factor: Molecular structure and implications for a central role in liver regeneration. J Gastroenterol Hepatol.

[b10] Marchand-Adam S, Fabre A, Mailleux AA, Marchal J, Quensel C, Kataoka H, Aubier M, Dehoux M, Soler P, Crestani B (2006). Defect of pro-hepatocyte growth factor activation by fibroblasts in idiopathic pulmonary fibrosis. Am J Respir Crit Care Med.

[b11] Miyazawa K, Shimomura T, Kitamura A, Kondo J, Morimoto Y, Kitamura N (1993). Molecular cloning and sequence analysis of the cDNA for a human serine protease responsible for activation of hepatocyte growth factor. Structure similarity of the protease precursor to blood coagulation factor XII. J Biol Chem.

[b12] Nakamura T, Teramoto H, Ichihara A (1986). Purification and characterization of a growth factor from rat platelets for mature parenchymal hepatocytes in primary cultures. Proc Natl Acad Sci USA.

[b13] Nayeri F, Nilsson I, Hagberg L, Brudin L, Roberg M, Söderström C, Forsberg P (2000). Hepatocyte growth factor levels in cerebrospinal fluid: A comparison between acute bacterial and nonbacterial meningitis. J Infect Dis.

[b14] Nayeri F, Brudin L, Nilsson I, Forsberg P (2002a). Sample handling and stability of hepatocyte growth factor in blood samples. Cytokine.

[b15] Nayeri F, Millinger E, Nilsson I, Zetterström O, Brudin L, Forsberg P (2002b). Exhaled breath condensate and serum levels of hepatocyte growth factor in pneumonia. Respir Med.

[b16] Nayeri F, Nilsson I, Brudin L, Fryden A, Söderström C, Forsberg P (2002c). High serum hepatocyte growth factor levels in the acute stage of community-acquired infections diseases. Scand J Infect Dis.

[b17] Nayeri F, Strömberg T, Larsson M, Brudin L, Söderström C, Forsberg P (2002d). Hepatocyte growth factor may accelerate healing in chronic legulcers: A pilot study. J Dermatol Treat.

[b18] Nayeri F, Olsson H, Peterson C, Sundqvist T (2004). Hepatocyte growth factor; expression, concentration and biological activity in chronic leg ulcers. J Dermatol Sci.

[b19] Nayeri F, Aili D, Nayeri T, Xu J, Almer S, Lundström I, Åkerlind B, Liedberg B (2005a). Hepatocyte growth factor (HGF) in fecal samples: Rapid detection by surface plasmon resonance. BMC Gastroenterol.

[b20] Nayeri F, Olsson H, Brudin L, Söderström C, Forsberg P, Peterson C, Sundqvist T (2005b). Hepatocyte growth factor in chronic leg ulcers—no biological activity—no improvement. JDS.

[b21] Nayeri F, Xu J, Abdiu A, Nayeri F, Aili D, Liedberg B, Carlsson U (2006). Autocrine production of biologically active hepatocyte growth factor (HGF) by injured human skin. JDS.

[b22] Nayeri F, Holmgren-Pettersson K, Perskvist N, Forsberg P, Peterson C, Sundqvist T (2007). An *in vitro* model for assessment of the biological activity of hepatocyte growth factor. Growth Factors.

[b23] Prat M, Narsimhan R, Crepaldi T, Nicotra M, Natali P, Comoglio P (1991). The receptor encoded by the human c-MET oncogene is expressed in hepatocytes, epithelial cells and solid tumors. Int J Cancer.

[b24] Rescan PY, Loreal O, Hassell JR, Yamada Y, Guillouzo A, Clement B (1993). Distribution and origin of the basement membrane component perlecan in rat liver and primary hepatocyte culture. Am J Pathol.

[b25] Stamos J, Lazarus R, Yao X, Kirchhofer D, Wiesmann C (2004). Crystal structure of the HGF β-chain in complex with the Sema domain of the Met receptor. EMBO J.

[b26] Stenberg E, Persson B, Roos H, Urbaniczky C (1991). Quantitative determination of surface concentration of protein with surface plasmon resonance using radiolabeled proteins. J Colloid Interface Sci.

[b27] Wang XS, Yip KH, Sam SW, Lau HY (2006). Buffy coat preparation is a convenient source of progenitors for culturing mature human mast cells. J Immunol Methods.

[b28] Wolf H, Zarnegar R, Michalopoulos GK (1991). Localization of hepatocyte growth factor in human and rat tissues: An immunohistochemical study. Hepatology.

[b29] Yamanouchi H, Fujita J, Yoshinouchi T, Hojo S, Kamei T, Yamadori I, Ohtsuki Y, Ueda N, Takahara J (1998). Measurement of hepatocyte growth factor in serum and bronchoalveolar lavage fluid in patients with pulmonary fibrosis. Respir Med.

